# Astragalean819: An Astragalean clade‐specific bait set to resolve phylogenetic relationships in *Astragalus*

**DOI:** 10.1002/aps3.70024

**Published:** 2025-10-03

**Authors:** Daniele Buono, Gudrun Kadereit, Diego F. Morales‐Briones

**Affiliations:** ^1^ Prinzessin Therese von Bayern‐Lehrstuhl für Systematik, Biodiversität & Evolution der Pflanzen, Ludwig‐Maximilians‐Universität München Munich Germany; ^2^ Staatliche Naturwissenschaftliche Sammlungen Bayerns (SNSB): Botanische Staatssammlung München, Botanischer Garten München‐Nymphenburg Munich Germany

**Keywords:** Astragalean clade, *Astragalus*, bait set, Fabaceae, herbarium, target enrichment

## Abstract

**Premise:**

*Astragalus* (Fabaceae) is the largest genus of flowering plants, with about 3100 species. Most phylogenies of the genus are based on a few nuclear or plastid loci (with one exception that uses ~100 loci) and usually provide poorly resolved trees and even conflicting subgeneric classifications. Target enrichment can greatly improve phylogenetic resolution, even at recently diverged taxonomic units, by generating sequences for hundreds of phylogenetically informative, putative single‐copy loci. However, a specific bait set for the Astragalean clade is still lacking.

**Methods:**

In this study, we use transcriptome data from 17 species in the Astragalean clade (of which eight are *Astragalus*) to identify putative single‐copy nuclear loci to build a bait set for target enrichment. This resulted in 819 exons from 686 genes across the Astragalean clade. The bait set was validated with a phylogenetic study based on 20 taxa selected across major clades in *Astragalus* plus three Astragalean species.

**Results:**

We recovered, on average, 739.3 loci covering at least 75% of the corresponding target reference length. The average number of paralog warnings was 76.2, ranging from 12 to 373. Our phylogenetic inference produced full support for all nodes, largely in agreement with the current subgeneric classifications of *Astragalus*.

**Discussion:**

Our bait set, Astragalean819, effectively retrieved highly informative loci to build a robust phylogeny that will help to clarify the complex relationships among members of the Astragalean clade and the subgeneric relationships in *Astragalus*. This study provides a foundation for advancing the understanding of the systematics and evolution of the largest flowering plant genus.

## TARGET ENRICHMENT

Currently, sequencing whole genomes is a state‐of‐the‐art technique that is a rich source of information. However, bioinformatic and computational challenges arise when analyzing datasets derived from large genomes, including many plant groups (Zhang et al., 2025). Furthermore, the effort and cost required to obtain whole‐genome data are often unnecessary in phylogenomic studies where an appropriate number of phylogenetically informative, single‐copy loci can be sufficient (Andermann et al., 2020). Using low‐copy or single‐copy nuclear (SCN) genes is highly beneficial for plant phylogenetics because they offer more rapidly evolving loci than the commonly used chloroplast and ribosomal sequences (Small et al., 2004; Zimmer and Wen, 2015). Target enrichment is one of several reduced‐representation strategies focusing on selected single‐copy loci across the genome (Mandel et al., 2014). The data obtained from those hundreds or even thousands of loci can resolve complex phylogenetic scenarios that involve processes such as incomplete lineage sorting or reticulate evolution. Target enrichment allows the sequencing effort to be focused on the desired loci, making this method particularly appropriate for museum and herbarium specimens, where highly degraded and fragmented DNA with sequences less than 100 bp is normally expected (McKain et al., 2018; Brewer et al., 2019; Forrest et al., 2019; Andermann et al., 2020). This method uses short RNA probes (usually 80–120 bp) that hybridize to complementary sequence library fragments (McKain et al., 2018). Hybridized fragments can be bound to magnetic beads and separated from the rest of the library, improving the depth of coverage for the genes of interest. This characteristic makes the method useful for analyzing specific variants, sequencing exomes and large numbers of genes, and inferring genome duplications and orthologous genes.

Probe design was a limitation of target enrichment in the past and was originally focused on highly conserved loci to achieve broad taxonomic coverage (Faircloth et al., 2012; Pezzini et al., 2023). However, the increasing availability of transcriptome sequence data across all major clades has facilitated the design of custom probe sets for specific plant groups (McKain et al., 2018; Vatanparast et al., 2018). Universal probe sets (e.g., Angiosperms353; Johnson et al., 2019) are developed to target mostly highly conserved loci, and while they are very useful in non‐model systems that lack a reference genome, they may not be able to provide enough data to resolve relationships in cases where introgression and low sequence divergence occur (Chau et al., 2018; Yardeni et al., 2022). When rapid radiation occurs, lineage‐specific probe sets can thus provide a higher amount of useful data to resolve shallow phylogenetic relationships (e.g., Eserman et al., 2021; Siniscalchi et al., 2021). Additionally, hybridization efficiency depends on the similarity between probes and target genomic regions (Cronn et al., 2012). Taxon‐specific probes, designed using genetic data obtained from a few species in the clade of interest, show higher performance compared to universal probe sets because they are less biased toward more conserved regions and provide higher resolution at a finer taxonomic scale, recovering more genomic variation (Pezzini et al., 2023).

### THE MEGA‐GENUS *ASTRAGALUS*

*Astragalus* L. (Fabaceae) belongs to the large Papilionoideae subfamily (~14,000 species; LPWG, 2017) in the so‐called inverted repeat–lacking clade (Wojciechowski et al., 2004). The genus is considered the largest in the flowering plants, with approximately 3100 species (POWO, 2024), which is about 800 more than the second‐largest genus, *Taraxacum* F. H. Wigg (Asteraceae; Moonlight et al., 2024). Most of the species (~2600) occur in the Eastern Hemisphere (Old World), and about 500 species are exclusive to the Western Hemisphere (New World) (Welsh, 2007; Podlech and Zarre, 2013). Although *Astragalus* has attracted considerable scientific attention and has been the focus of extensive research, a well‐supported phylogeny and comprehensive explanation for its remarkable diversity remain elusive. Numerous attempts have been made to resolve the backbone phylogeny of the genus using various molecular markers. Among the most recent studies, Azani et al. (2017, 2019) built a representative phylogeny (comprising 150 and 189 *Astragalus* species, respectively) of most clades in the Eastern Hemisphere based on nuclear ITS and plastid *trnK/matK*. In the present study, we follow the clade nomenclature established by Azani et al. (2017), who identified 11 well‐supported major clades within a monophyletic Eu‐*Astragalus*, which includes the Glottis clade (comprising the genus *Biserrula* L.) and *Astragalus* sensu stricto. Despite this framework, their phylogeny suffered from poor resolution and several polytomies at shallow levels (Azani et al., 2017). Analyses of whole‐plastome sequences have also been attempted (e.g., Su et al., 2021); however, those often produce discordant topologies compared to nuclear phylogenies (Soltis and Kuzoff, 1995; Záveská et al., 2019; Maylandt et al., 2024).

Folk et al. (2024) reconstructed the largest phylogeny of the genus so far based on a selection of 100 loci designed to target sequences of the whole nitrogen‐fixing clade (Soltis et al., 1995) as part of the NitFix sequencing initiative (Kates et al., 2024). This notable effort included around 850 *Astragalus* species, including ~370 species exclusive to the Western Hemisphere, known as Neo‐*Astragalus*, and ~470 Eastern Hemisphere species. Their results, however, were hindered by poor resolution, especially at shallow nodes, particularly in Neo‐*Astragalus*. Even though the main clades recovered by Folk et al. (2024) overlapped partially with previous phylogenetic studies (Azani et al., 2017; Su et al., 2021), many nodes still conflicted with them (e.g., a paraphyletic Eu‐*Astragalus* with the segregation of the *Biserrula* genus from *Astragalus*, or Neo‐*Astragalus* being sister to the Hypoglottis and Diholcos clades). Given its remarkable species richness, widespread distribution across semi‐arid regions of the Northern Hemisphere, and exceptionally high diversification rates (Folk et al., 2024), *Astragalus* represents an ideal model clade for investigating phylogenetic diversity at shallow evolutionary scales (Cavender‐Bares, 2019). However, the absence of a comprehensive and well‐supported phylogeny remains a major limitation for studies seeking to uncover the processes driving its lineage diversification.

### OBJECTIVES

In this study, we had two goals: (1) to investigate the DNA quality obtained from historical to recent herbarium specimens of *Astragalus* and its usability for high‐throughput sequencing, and (2) to investigate the capability of a custom bait set for target enrichment, Astragalean819, to build a robust phylogeny of *Astragalus*, focusing on relationships at the subgeneric level. In regard to the first goal, plant material from herbarium collections is an invaluable resource when studying large groups, especially when the sampling areas are logistically or politically inaccessible (e.g., for *Astragalus*; Folk et al., 2024). Here, we leveraged the large, near‐complete *Astragalus* collection of the Botanische Staatssammlung München herbarium, which includes about 22,000 specimens with a worldwide distribution. For our second aim, the Astragalean819 bait set included 819 putative single‐copy loci derived from transcriptome data of Astragalean taxa. We expected that the combination of a large number of nuclear targets and taxon‐specific probe design would deliver the first fully resolved backbone phylogeny for the genus. Custom bait sets like this one are increasingly recognized as powerful tools in evolutionary biology, enabling high‐resolution phylogenomic analyses even from degraded or historical DNA sources. Their broad applicability across related taxa offers enormous potential for addressing questions of diversification, biogeography, and trait evolution at various taxonomic scales. Target enrichment was tested using genomic DNA extracted from herbarium specimens as old as 95 years, representing all of the sections currently supported by molecular data in the genus, as well as three species from other genera within the Astragalean clade.

## METHODS

### Bait set design

To design our custom bait set for the Astragalean clade (sensu Sanderson and Wojciechowski, 1996), we identified putative SCN genes using MarkerMiner v.1.2 (Chamala et al., 2015) with default settings, except that the minimum transcript length was set to 500 bp. We used transcriptomes of 17 species (eight from representatives of *Astragalus* and nine from other genera across the Astragalean clade; Zhao et al., 2021) (Appendix S1, see Supporting Information with this article), as well as the genomes of *Cicer arietinum* L. (Varshney et al., 2013), *Medicago truncatula* Gaertn. (Tang et al., 2014), and *Trifolium pratense* L. (De Vega et al., 2015) as references. MarkerMiner was run individually using the same parameters for each of the three reference genomes. SCN genes identified with MarkerMiner were further filtered and split into exons using GoldFinder (Vargas et al., 2019), requiring genes with a minimum length of 500 bp and coverage of at least three species. This resulted in 1361, 1414, and 1276 filtered genes using *C. arietinum*, *M. truncatula*, and *T. pratense* as reference, respectively. To avoid potential chimeric sequences from paralogous copies during the assembly of multi‐exon genes (Morales‐Briones et al., 2018), we selected loci on an exon rather than a gene basis. If loci selection resulted in several exons of the same gene, we did not assemble them together; rather, we treated each exon as its own evolutionary unit for phylogenetic analysis. For exon selection, GoldFinder results for each MarkerMiner run were first merged using the *Arabidopsis thaliana* (L.) Heynh. gene names, resulting in 2109 shared and unique genes identified from each reference. Individual exon alignments from those 2109 genes were first filtered to keep only those of at least 500 bp, resulting in 3359 exons from 929 genes. Individual exon FASTA files were realigned using MACSE v.2.06 (Ranwez et al., 2018) to correct codon frames and visually inspected in GENEIOUS v.11.1.5 (https://www.geneious.com) to remove potential misassemblies from the starts or ends of the alignments. Additionally, exon alignments were filtered to include only those at least 500 bp in length and with pairwise identity between 75% and 98% across the alignment. Final exon selection was done by prioritizing identical shared exons among all three references, resulting in 819 exons from 686 genes across the Astragalean clade. For bait design, we filtered the 819 exon alignments to include only two sequences, one from a species of *Astragalus* and one from a species of any other genus within the Astragalean clade. The custom set of 80‐bp biotinylated RNA baits (myBaits) was manufactured by Daicel Arbor Biosciences (Ann Arbor, Michigan, USA) with a 2× tiling density, and the final kit included 36,572 baits. To assess the overlap with the “universal” Angiosperms353 bait set, we identified the corresponding *Arabidopsis thaliana* gene names reported by Johnson et al. (2019) and matched them with the gene names from the output of MarkerMiner. Similarly, we identified loci shared with the NitFix bait set (Kates et al., 2024) by manually searching the identical sequences from the NitFix target file into the *Arabidopsis thaliana* coding DNA sequence file provided by MarkerMiner.

### DNA isolation, library preparation, and sequencing

We selected 23 species to represent the 10 major clades recognized in *Astragalus* by Azani et al. (2017), Su et al. (2021), and Folk et al. (2024). Herbarium specimens were preferred based on recency, certainty of determination, and visual assessment of preservation (greener specimens were favored). DNA was extracted from ~20 mg of dry herbarium plant material using the NucleoSpin Plant II kit (Macherey‐Nagel, Düren, Germany), following the manufacturer's manual with the following modifications: (1) in step 2a, cell lysis was performed using 600 μL of buffer PL1, no RNase A was added because the plant material was too old to preserve significant amounts of RNA (which would have interfered with the next steps), and incubation lasted for 1.5 h; (2) in step 6, an extra washing was performed, adding 350 μL of buffer PW2 before drying the membrane completely; and (3) DNA was eluted in 50 μL of buffer PE (5 mM Tris/HCl, pH 8.5). DNA concentration was measured using an Invitrogen Qubit 4 Fluorometer with the High Sensitivity (HS) assay kit (Thermo Fisher Scientific, Waltham, Massachusetts, USA), and fragmentation was visually evaluated on a 1% agarose gel. For all samples, regardless of the fragmentation observed on the agarose gel, genomic DNA was diluted to 250 ng in 55 μL of buffer PE and sonicated using the same program with a Covaris M220 Focused‐ultrasonicator (Covaris, Woburn, Massachusetts, USA) to obtain DNA fragments of approximately 350 bp. We accurately profiled the sonicated sample size distribution using an Agilent 4150 TapeStation System with the High Sensitivity D1000 ScreenTape (Agilent Technologies, Santa Clara, California, USA). Libraries were prepared using the NEBNext Ultra II DNA Library Prep Kit for Illumina and the NEBNext Multiplex Oligos for Illumina (Dual Index Primers Set 1; New England Biolabs, Ipswich, Massachusetts, USA), following the manufacturer's protocol. Because we used ~250 ng of starting DNA, we performed a size selection of adapter‐ligated DNA (step 3 of the protocol), adjusting by the average sample size measured with TapeStation for each sample, as recommended by the manufacturer's protocol. The adapter‐ligated DNA was amplified with eight PCR cycles, and each library was profiled using the Qubit and TapeStation, as described above.

Individual libraries were mixed in 16 sample pools (the second pool included additional libraries, which were used in another study) using the same DNA amounts (250 ng) for each library and pooling together libraries with similar average fragment sizes (ranging from 277 to 526 bp). Pooled libraries were dried by vacuum centrifugation and then resuspended in 7 μL of nuclease‐free water. Target enrichment was performed using the myBaits Hybridization Capture Kits (Arbor Biosciences), following the manufacturer's protocol (v.5.02). The hybridization temperature (*T*_H_) and wash temperature (*T*_W_) were both set at 60°C. The enriched libraries were amplified with 10 PCR cycles. After amplification, the pooled libraries were purified using the NucleoSpin Gel and PCR Clean‐up Kit (Macherey‐Nagel) and characterized by measuring concentration and fragment sizes using the Qubit and TapeStation. The two enriched pools were again pooled in equimolar quantities (7 nM) and sequenced at the Core Facility Genomics of the Helmholtz Zentrum München, Germany (Deutsches Forschungszentrum für Gesundheit und Umwelt). Sequencing was performed on an Illumina NextSeq 1000 sequencing system with 150‐bp paired‐end reads, aiming for 1000× coverage per sample.

### Assembly and phylogenetic inference

Raw read quality control was performed using FastQC v.0.11.8 (Andrews, 2010) and MultiQC v.1.19 (Ewels et al., 2016). Duplicated reads (identical or nearly identical sequences with some mismatches from PCR) were removed using ParDRe v.2.2.5, with the default setting of zero allowed mismatches (González‐Domínguez and Schmidt, 2016). To cut sequencing adapters and poor‐quality sequences, we used Trimmomatic v.0.39 (parameters: ILLUMINACLIP: TruSeq_ADAPTER: 2:30:10 SLIDINGWINDOW: 4:5 LEADING: 5TRAILING: 5 MINLEN: 25) (Bolger et al., 2014). Quality control of deduplicated and clean reads was done with FastQC and MultiQC, and loci were assembled with HybPiper v.2.1.5 (Johnson et al., 2016). The percentage similarity threshold for the sliding window (‐‐exonerate_hit_sliding_window_thresh option) and the percent identity threshold for retaining Exonerate hits (‐‐thresh option) were set to 85, the option ‐‐chimeric_stitched_contig_edit_distance was set to 0, and ‐‐chimeric_stitched_contig_discordant_reads_cutoff was set to 1. The target file (option ‐t_dna) consisted of all the Astragalean sequences for 819 loci used for bait design. We used Diamond v.2.1.8 (Buchfink et al., 2021) to map reads to the target loci and used the *paralog_retriever* function included in HybPiper to obtain coding sequences from putative alternative long paralogs. Sequences were aligned using MACSE v.2.07 (Ranwez et al., 2018), and AMAS was used to assess the informativeness of individual loci (Borowiec, 2016). To remove sites that showed missing data, we used Pxclsq v.1.3 (Brown et al., 2017) by setting the minimum column occupancy to 0.1 (10%). Gene trees were inferred using IQ‐TREE v.2.3.0 (Minh et al., 2020) with standard model selection for ModelFinder (option ‐m TEST; Kalyaanamoorthy et al., 2017) and 1000 Ultrafast bootstrap replicates for node support. We masked tips in the gene trees that were either monophyletic or paraphyletic, and retained the tip with the most unambiguous characters in the trimmed alignment (Yang et al., 2015). We used TreeShrink v.1.3.9 (Mai and Mirarab, 2018) to remove abnormally long branches using a quantile value of 0.1 and excluding outgroups. Final homologous FASTA files were generated from those trees and aligned using OMM MACSE v.12.01 (Ranwez et al., 2018). Homologous gene trees were then generated from those sequences using IQ‐TREE with the same settings as above. To infer a coalescent‐based species tree, we used ASTRAL‐Pro3 v1.19.3.5 (Zhang and Mirarab, 2022), using the final homologous trees as input, as this tool allows for multi‐copy genes, and setting the option ‘‐‐support 2’ to obtain local posterior probabilities (LPP) and quartet scores for each internal node. To estimate the amount of off‐target chloroplast data in our raw reads, we used Fast‐Plast v.1.2.9 (McKain and Wilson, 2017) and attempted to assemble plastome sequences, using default settings and Fabales for read mapping (‐‐bowtie_index). A more detailed phylogenetic analysis was performed by Buono et al. (2025a), including additional taxa.

## RESULTS

### Selected loci and bait set testing

**Figure 1:**
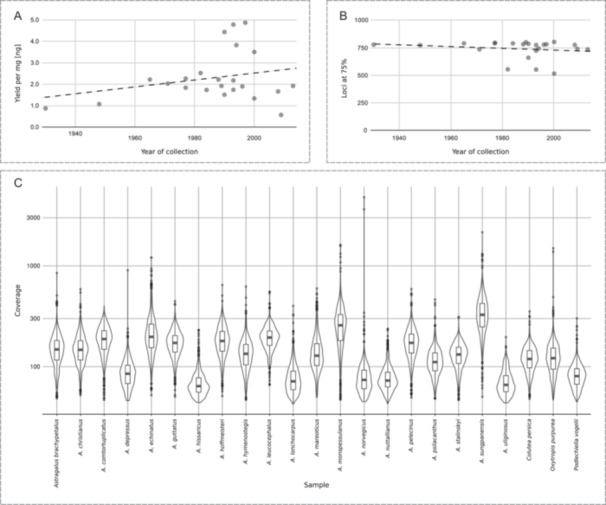
Genomic DNA and targeted genes recovered from herbarium material. (A) Scatter plot showing genomic DNA yield obtained from one milligram of dry leaf compared with specimen age. A weak trend was observed (dashed line, *R* ^2^ = 0.067). (B) Scatter plot showing loci recovered with at least 75% of the target sequence length vs. specimen age. A non‐significant effect of age on the loci recovered was observed (*R* ^2^ = 0.033). (C) Violin plot showing average mapped locus coverage per sample. A logarithmic scale for coverage was used to enhance the clarity of the plot.

A list of the 686 genes included in the Astragalean819 bait set is provided in Appendix S2. The 819 exons belonging to those 686 genes had a cumulative length (capture space) of 796,855 bp, with an average exon length of 972.96 bp (ranging from 417 to 3798 bp). We obtained an overlap of 43 loci with Angiosperms353 and 88 loci with NitFix (Appendix S2). DNA extractions were successful for all 23 specimens tested, with yields ranging from 650 to 5600 ng of genomic DNA (Figure 1A, Appendix S3). Fragmentation was assessed on agarose gel (results not shown) and was very high, especially for the oldest specimens (the oldest being from 1930; Appendix S3). Nevertheless, profiles of the enriched libraries matched the expected size (ranging from ~200 to ~600 bp, with peaks around 350 bp; results not shown) and concentration (~11 and ~16 ng/µL). Sequencing was successful for all samples, with raw sequences ranging between 2.8 million and 13.8 million.

### Assembly and alignment statistics

**Figure 2:**
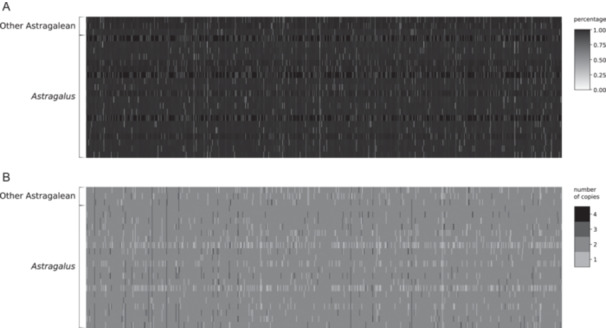
Heatmaps showing the results of the HybPiper assembly. (A) The length of the retrieved loci is represented as the percentage relative to the reference. (B) Number of paralogs flagged in each locus.

**Figure 3:**
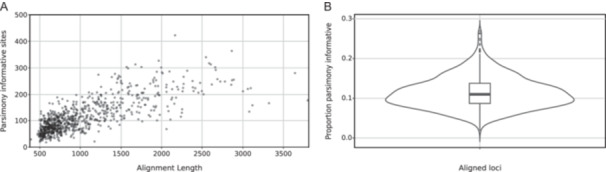
Parsimony‐informative sites in sequences retrieved by HybPiper. (A) Scatter plot showing the number of parsimony‐informative sites against the alignment length. (B) Violin plot showing the distribution of the proportion of parsimony‐informative sites across all aligned loci (average 11.4%, ranging from 1.8% to 26.9%).

HybPiper assembly statistics are summarized in Figure 2 and Table 1. We obtained high percentages of targeted genes (Figure 2A), and all 819 targeted loci were mapped on the reads for all samples (Table 1). On average, across all samples, we obtained 9,515,168.9 reads (4,757,584 paired reads, as we used paired‐end reads), of which 2,184,065.7 (23.0%) were mapped to sequences using DIAMOND (ranging from 13.4% for *A. norvegicus* Weber to 38.5% for *A. sungpanensis* E. Peter; Table 1). No strong correlation was found between the number of raw reads sequenced and the age of the specimens (*R* ^2^ = 0.078; Appendix S4). This resulted in an average locus depth of coverage higher than 100× for most samples (Figure 1C). The average number of loci recovered with sequences more than 75% of the mean target length (i.e., the number of exons with sequences >75% of the target length) was 739.3, ranging from 517 (*A. uliginosus* L.) to 805 (*A. leucocephalus* Graham ex Benth.) (Table 1, Figure 1B). We observed a negligible effect of sample age on the number of loci at 75% (*R* ^2^ = 0.033; Figure 1B). We obtained, on average, 76.2 paralog warnings per sample, based on sequencing depth, ranging from 12 (*A. hissaricus* Lipsky, *A. norvegicus*, *A. uliginosus*) to 373 (*A. echinatus* Murray; Table 1, Figure 2B). Finally, on average, the total number of bases recovered was 727,663.0, ranging from 534,834 (*A. uliginosus*) to 787,215 (*A. sungpanensis*), with sequences retrieved from all 819 exons belonging to 686 genes. We were able to assemble partial plastome sequences from off‐target regions using Fast‐Plast, obtaining, on average, 64.5% of the known angiosperm chloroplast gene sequences across samples, ranging from 3.7% (*A. monspessulanus* L.) to 80.2% (six samples; Appendix S5). Alignment statistics obtained with AMAS are summarized in Figure 3 (for complete results, see Appendix S6). On average, across loci, we observed 6.8% of missing data (ranging from 0% to 40.8%). The number of variable sites per locus ranged from 63 to 873 (average 252.8), with an average of 113.7 parsimony‐informative sites (ranging from 18 to 423). This resulted in an average of 11.4% parsimony‐informative sites across all loci (ranging from 1.8% to 26.9%).

**Table 1:** Summary of HybPiper assembly statistics.

Species	No. of reads	No. of reads on target (%)	No. of exons with reads	No. of exons with sequences	No. of exons with sequences >50% of the target length	No. of exons with sequences >75% of the target length	No. of exons with paralog warning based on length	No. of exons with paralog warning based on depth	Total no. of bases recovered
*Astragalus brachypetalus*	13,096,388.00	2,197,494 (16.8%)	819	794	790	780	28	43	757,905
*A. christianus*	10,890,516.00	2,162,531 (19.9%)	819	790	784	775	21	30	761,679
*A. contortuplicatus*	12,954,696.00	2,476,545 (19.1%)	819	798	795	792	23	25	771,726
*A. depressus*	3,803,474.00	1,081,395 (28.4%)	819	759	754	738	16	18	722,751
*A. echinatus*	21,947,158.00	4,067,973 (18.5%)	819	804	801	796	287	373	772,437
*A. guttatus*	14,311,544.00	2,414,407 (16.9%)	819	798	796	794	16	18	771,000
*A. hissaricus*	5,194,436.00	818,111 (15.7%)	819	613	594	556	11	12	578,916
*A. hoffmeisteri*	6,693,262.00	2,560,090 (38.2%)	819	796	794	792	17	20	781,161
*A. hymenostegis*	8,930,610.00	1,986,014 (22.2%)	819	797	793	784	36	42	761,667
*A. leucocephalus*	13,467,798.00	2,747,841 (20.4%)	819	805	805	802	19	20	777,093
*A. lonchocarpus*	5,926,928.00	1,440,551 (24.3%)	819	728	706	662	216	253	680,127
*A. mareoticus*	9,754,798.00	2,638,608 (27%)	819	805	804	788	239	293	763,911
*A. monspessulanus*	12,255,330.00	4,235,194 (34.6%)	819	786	782	777	24	30	758,790
*A. norvegicus*	6,846,986.00	918,944 (13.4%)	819	593	580	554	9	12	569,157
*A. nuttallianus*	5,466,098.00	1,430,111 (26.2%)	819	763	758	726	300	354	719,895
*A. pelecinus*	8,200,056.00	2,252,069 (27.5%)	819	752	750	745	13	13	721,428
*A. psilacanthus*	4,518,478.00	1,592,032 (35.2%)	819	792	791	781	23	31	770,313
*A. stalinskyi*	8,108,134.00	1,800,468 (22.2%)	819	789	788	784	19	20	755,565
*A. sungpanensis*	15,393,230.00	5,932,392 (38.5%)	819	814	811	805	29	64	787,215
*A. uliginosus*	4,429,540.00	789,492 (17.8%)	819	544	537	517	10	12	534,834
*Colutea persica*	7,406,784.00	1,663,545 (22.5%)	819	791	787	778	26	31	759,198
*Oxytropis purpurea*	12,328,132.00	1,824,155 (14.8%)	819	756	752	742	13	23	729,177
*Podlechiella vogelii*	6,924,508.00	1,203,549 (17.4%)	819	746	746	737	16	16	730,305
**Average**	**9,515,168.9**	**2,184,065.7 (23.0%)**	**819.0**	**757.1**	**752.1**	**739.3**	**61.3**	**76.2**	**727,663.0**
**Sum**	**218,848,884.0**	**50,233,511.0**							

### Phylogenetic inference

**Figure 4:**
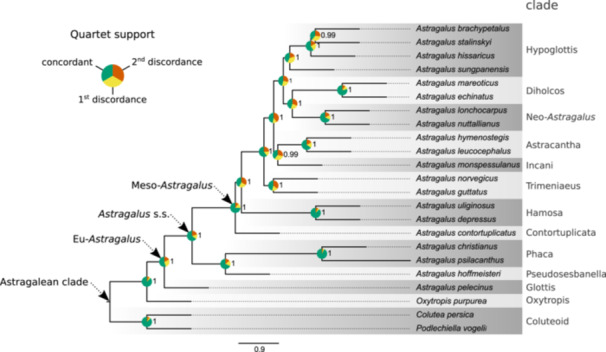
Coalescent species tree inferred by ASTRAL‐Pro based on 819 homologous gene trees. Numbers to the right of nodes indicate local posterior probability. Pie charts at internal nodes indicate the percentage of quartets in gene trees that agree with the shown main topology (green; average 60.6%, minimum 39.3%, maximum 92.6%), the first alternative topology (yellow; average 18.6%, minimum 3.9%, maximum 36.9%), and the second alternative topology (orange; average 20.8%, minimum 3.5%, maximum 39.4%). Section names follow Azani et al. (2017).

We obtained a coalescent‐based phylogeny using ASTRAL‐Pro3 from 819 homologous gene trees, which was fully supported (LPP = 1) for all but two nodes (LPP = 0.99) (Figure 4). Quartet topologies were mostly concordant with the species tree for most of the nodes, with an average of 60.6% (ranging from 39.3% to 92.6%) for the concordant topology and 18.6% (ranging from 3.8% to 36.9%) and 20.8% (ranging from 3.5% to 39.4%) for the two discordant alternatives. We recovered a monophyletic Eu‐*Astragalus*, comprising all *Astragalus* species included in 11 clear infrageneric clades, albeit with a very limited number of taxa to represent them, which exhibited a strong overlap with previous studies (Azani et al., 2017; Su et al., 2021; Folk et al., 2024).

## DISCUSSION

In the present work, we explored the possibility of obtaining a strong phylogenetic signal to resolve subgeneric relationships in *Astragalus* by using genomic DNA obtained from herbarium material and an Astragalean‐specific bait set for target enrichment. Herbarium specimens were the sole source of genomic DNA, and despite some specimens being more than 50 years old (notably, *A. nuttallianus* DC. was collected in 1930), we were able to obtain a sufficient amount of genomic DNA (with a minimum of 650 ng). There was no apparent correlation between age and genomic DNA yield (Figure 1A), although our sampling was biased towards more recent specimens, with only a few (26%) collected before 1980. The observed high fragmentation (results not shown) was expected (McKain et al., 2018; Brewer et al., 2019; Forrest et al., 2019; Andermann et al., 2020); nevertheless, there was no apparent negative trend between specimen age and the number of loci recovered (Figure 1B). Additionally, because the bait set was designed using transcriptomes from several Astragalean taxa, no differences were observed between *Astragalus* species and the three Astragalean species in terms of genomic DNA yield and loci recovered (Table 1).

In *Astragalus*, many phylogenetic studies have relied on a few nuclear/plastid markers, such as ITS and *matK* (e.g., Kazempour Osaloo et al., 2005; Dizkirici et al., 2014; Azani et al., 2017, 2019; Duan et al., 2024), which have the advantage of being quite conserved among taxa and thus easy to amplify and sequence using Sanger sequencing (Baldwin et al., 1995; Shaw et al., 2014). Additionally, because of their ubiquitous usage, many sequences are available online, including for many *Astragalus* species. On the other hand, their conserved nature becomes a disadvantage when the goal is to obtain a sufficient resolution to resolve a shallow phylogeny (i.e., at low taxonomic levels or rapidly diverging lineages) (Small et al., 2004). As a result, the attempts to produce a strongly supported and well‐resolved backbone phylogeny in *Astragalus* using ITS/*matK* have been unsuccessful, probably also due to the relatively recent radiation of the genus ~16 Mya (12.27–20.76 Mya; Azani et al., 2019). Moreover, whole‐plastome phylogenies, like the one produced by Su et al. (2021), are limited because plastomes evolve in a manner similar to a single gene (Doyle, 2022). Additionally, while biparental plastid inheritance has been observed in some legumes (Rajora and Mahon, 1995; Zhang et al., 2003; Matsushima et al., 2008), the mode of organellar inheritance in *Astragalus* is still unknown. The higher level of sequence variation in low‐copy nuclear loci makes their use preferable compared to organellar genes, which are biparentally inherited (Small et al., 2004). In this context, target enrichment offers an easy way to obtain multiple single‐copy loci across the whole genome of Astragalean species.

Loci with high variability, low number of paralogs, and long average length are desirable for phylogenomics (McKain et al., 2018; Ning et al., 2024). Although our alignment statistics showed high sequence divergence and informativeness (Figure 3), we obtained paralog warnings from HybPiper based on depth for several samples (Table 1). Interestingly, the most paralog warnings were generated for species belonging to the Diholcos clade (*A. mareoticus* Delile and *A. echinatus*) and the Neo‐*Astragalus* clade (*A. lonchocarpus* Torr. and *A. nuttallianus*) (Table 1). The paralogy observed can be attributed to whole‐genome duplication events across the phylogeny of Fabaceae (e.g., Zhao et al., 2021). In a study by Folk et al. (2024) designed to target taxa in the whole nitrogen‐fixing clade (NitFix; Kates et al., 2024), the low phylogenetic resolution may have been caused by high levels of paralogy resulting from the use of a more general bait set in a group where polyploidy is quite common. Overall, plants exhibit a high prevalence of paralogous genes, and a proper evaluation of their effect and orthology inference is necessary (Morales‐Briones et al., 2022). A clade‐specific bait set facilitates orthology inference more effectively than a more general or universal one. Evaluation of the paralogy in our dataset is explored in Buono et al. (2025a) as it goes beyond the scope of this study; however, here we used a coalescent‐based method that addresses paralogy and orthology during tree inference.

We obtained a high number of targeted sequences per sample (Table 1). Additionally, by using off‐target sequences, we were able to obtain chloroplast sequences (Appendix S5), similar to studies on other plant groups (e.g., Baldwin et al., 2023; Bratzel et al., 2023). Even though we could not assemble entire plastomes, the sequences recovered may still contain enough information to build a robust phylogeny (Granados‐Mendoza et al., 2020; Bentz and Leebens‐Mack, 2024). Based on their different recombination dynamics, the recovered plastome sequences should be treated as a single gene using a concatenated approach, rather than as an average multi‐intron gene (Doyle, 2022). The usability of plastome sequences for phylogenetic inference is evaluated in a separate study (Buono et al., 2025a).

The coalescent‐based species tree inferred with Astral‐Pro based on 819 homologous gene trees had full support for all nodes (LPP ≥ 0.99; Figure 4). Additionally, the topology we obtained largely aligns with previous works (Su et al., 2021; Folk et al., 2024), and there is a close overlap between the clades recovered and those proposed by Azani et al. (2017). Among the most significant discrepancies we observed with Folk et al. (2024) are the placement of Neo‐*Astragalus* taxa inside the Diholcos clade instead of as an independent clade and the inclusion of the Glottis clade (here represented by *Astragalus pelecinus* (L.) Barneby) inside Eu‐*Astragalus*. The placement of those two clades in our tree instead aligns with the results of Azani et al. (2017). To better understand intrageneric relationships within *Astragalus*, such as the placement of the Glottis clade, a much denser sampling across clades is required (see Buono et al., 2025a). Nonetheless, our current results demonstrate that Astragalean819 has the potential to obtain a robust backbone phylogeny and provide enough data to analyze complex relationships within the genus. We estimated an overlap of 88 loci with NitFix and 43 loci with Angiosperms353 (Appendix S2). This overlap suggests that, once missing data are accounted for, newly produced Astragalean819 data can be combined with publicly available datasets from previous studies, thereby significantly increasing the sampling size, as shown in previous studies (Siniscalchi et al., 2021). A final consideration regarding our sequencing effort is the decision to target approximately 1000× coverage for the enriched libraries. Although such high coverage likely exceeded what was strictly necessary to recover all targeted loci, it proved advantageous in complex cases. Specifically, previous work has demonstrated that higher sequencing depth facilitates the successful assembly of multiple paralogous copies in groups with histories of whole‐genome duplications (Morales‐Briones et al., 2022). Therefore, determining the appropriate sequencing depth in future studies should be guided by the complexity of the clade and the specific research goals.

With the present work, we demonstrated that (1) herbarium material is a reliable source of genomic DNA for reconstructing *Astragalus* phylogeny using target enrichment methods, as already shown by Folk et al. (2024), and (2) the 819 putative single‐copy loci targeted by Astragalean819 contain strong phylogenetic information, enabling the recovery of a robust phylogeny of *Astragalus*. The bait set we developed represents a valuable resource for future research on the evolutionary history of the largest genus of flowering plants. Because the probes were designed from representatives across the whole Astragalean clade, they are broadly transferable and will facilitate phylogenomic and macro‐evolutionary studies throughout the group, opening new opportunities to explore diversification, biogeography, and trait evolution, while unlocking the vast and underutilized genomic potential stored in herbarium collections worldwide.

## AUTHOR CONTRIBUTIONS

D.F.M.B. and G.K. conceived the idea. D.B. and D.F.M.B. performed the experiments and conducted formal analyses. All authors contributed to the scientific discussion and the final manuscript draft, and all authors approved the final version of the manuscript.

## Supporting information


**Appendix S1:** Transcriptomes of species belonging to the Astragalean clade generated by Zhao et al. ( 2021) and utilized for bait design in this study.
**Appendix S2:** List of genes included in the Astragalean819 bait set. Reference gene names correspond to *Cicer arietinum* (CA), *Medicago truncatula* (MT), and *Trifolium pratense* (TP) used as reference. The respective gene name for *Arabidopsis thaliana* (AT) is provided. The inclusion of the same genes in Angiosperms353 (Johnson et al., 2019) and NitFix (Kates et al., 2024) is indicated.
**Appendix S3:** Voucher information of samples and results of genomic DNA extractions.

**Appendix S4:** Variation in the number of raw reads sequenced from each specimen according to specimen age. The dashed line represents the trend line (*R*² = 0.078).
**Appendix S5:** Summary of plastome assembly statistics.
**Appendix S6:** AMAS statistics produced from raw homologous sequences.

## Data Availability

Target enrichment data generated for this study can be found in the National Center for Biotechnology Information (NCBI) BioProject PRJNA1242075 (SRA accession numbers are provided in Appendix S3). The bait sequence and assembly reference, plus all the trees generated in this study, are available at the Dryad repository (Buono et al., 2025b; https://doi.org/10.5061/dryad.79cnp5j7g).

## References

[aps370024-bib-0001] Andermann, T. , M. F. Torres Jiménez , P. Matos‐Maraví , R. Batista , J. L. Blanco‐Pastor , A. L. S. Gustafsson , L. Kistler , et al. 2020. A guide to carrying out a phylogenomic target sequence capture project. Frontiers in Genetics 10: e1407. 10.3389/fgene.2019.01407 PMC7047930 32153629

[aps370024-bib-0002] Andrews, S. 2010. FastQC: A quality control tool for high throughput sequence data. Website: http://www.bioinformatics.babraham.ac.uk/projects/fastqc/ [accessed 8 September 2025].

[aps370024-bib-0003] Azani, N. , A. Bruneau , M. F. Wojciechowski , and S. Zarre . 2017. Molecular phylogenetics of annual *Astragalus* (Fabaceae) and its systematic implications. Botanical Journal of the Linnean Society 184(3): 347–365.

[aps370024-bib-0004] Azani, N. , A. Bruneau , M. F. Wojciechowski , and S. Zarre . 2019. Miocene climate change as a driving force for multiple origins of annual species in *Astragalus* (Fabaceae, Papilionoideae). Molecular Phylogenetics and Evolution 137: 210–221. 31102688 10.1016/j.ympev.2019.05.008

[aps370024-bib-0005] Baldwin, B. G. , M. J. Sanderson , J. M. Porter , M. F. Wojciechowski , C. S. Campbell , and M. J. Donoghue . 1995. The ITS region of nuclear ribosomal DNA: A valuable source of evidence on angiosperm phylogeny. Annals of the Missouri Botanical Garden 82: 247–277.

[aps370024-bib-0006] Baldwin, E. , M. McNair , and J. Leebens‐Mack . 2023. Rampant chloroplast capture in *Sarracenia* revealed by plastome phylogeny. Frontiers in Plant Science 14: e1237749. 10.3389/fpls.2023.1237749 PMC10497973 37711293

[aps370024-bib-0007] Bentz, P. C. , and J. Leebens‐Mack . 2024. Developing Asparagaceae1726: An Asparagaceae‐specific probe set targeting 1726 loci for Hyb‐Seq and phylogenomics in the family. Applications in Plant Sciences 12(5): e11597. 39360194 10.1002/aps3.11597 PMC11443443

[aps370024-bib-0008] Bolger, A. M. , M. Lohse , and B. Usadel . 2014. Trimmomatic: A flexible trimmer for Illumina sequence data. Bioinformatics 30(15): 2114–2120. 24695404 10.1093/bioinformatics/btu170 PMC4103590

[aps370024-bib-0009] Borowiec, M. L. 2016. AMAS: A fast tool for alignment manipulation and computing of summary statistics. PeerJ 4: e1660. 26835189 10.7717/peerj.1660 PMC4734057

[aps370024-bib-0010] Bratzel, F. , J. Paule , J. Leebens‐Mack , E. M. Leme , R. C. Forzza , M. A. Koch , S. Heller , and G. Zizka . 2023. Target‐enrichment sequencing reveals for the first time a well‐resolved phylogeny of the core Bromelioideae (family Bromeliaceae). Taxon 72(1): 47–63.

[aps370024-bib-0011] Brewer, G. E. , J. J. Clarkson , O. Maurin , A. R. Zuntini , V. Barber , S. Bellot , N. Biggs , et al. 2019. Factors affecting targeted sequencing of 353 nuclear genes from herbarium specimens spanning the diversity of angiosperms. Frontiers in Plant Science 10: e1102. 10.3389/fpls.2019.01102 PMC6759688 31620145

[aps370024-bib-0012] Brown, J. W. , J. F. Walker , and S. A. Smith . 2017. Phyx: Phylogenetic tools for unix. Bioinformatics 33(12): 1886–1888. 28174903 10.1093/bioinformatics/btx063 PMC5870855

[aps370024-bib-0013] Buchfink, B. , K. Reuter , and H. Drost . 2021. Sensitive protein alignments at tree‐of‐life scale using DIAMOND. Nature Methods 18(1): 1265–1272. 10.1038/s41592-021-01101-x PMC8026399 33828273

[aps370024-bib-0014] Buono, D. , G. Kadereit , A. Liston , S. Zarre , and D. F. Morales‐Briones . 2025a. Building a robust backbone for *Astragalus* using a clade‐specific target enrichment bait set. American Journal of Botany 112(8): e70084. 40827654 10.1002/ajb2.70084 PMC12374571

[aps370024-bib-0015] Buono, D. , G. Kadereit , A. Liston , S. Zarre , and D. F. Morales‐Briones . 2025b. Data from: Building a robust backbone for *Astragalus* (Fabaceae) using a clade‐specific target enrichment bait set. Dryad Dataset 10.5061/dryad.79cnp5j7g [accessed 12 September 2025]. PMC12374571 40827654

[aps370024-bib-0016] Cavender‐Bares, J. 2019. Diversification, adaptation, and community assembly of the American oaks (*Quercus*), a model clade for integrating ecology and evolution. New Phytologist 221(2): 669–692. 30368821 10.1111/nph.15450

[aps370024-bib-0017] Chamala, S. , N. García , G. T. Godden , V. Krishnakumar , I. E. Jordon‐Thaden , R. De Smet , W. B. Barbazuk , et al. 2015. MarkerMiner 1.0: A new application for phylogenetic marker development using angiosperm transcriptomes. Applications in Plant Sciences 3(4): e1400115. 10.3732/apps.1400115 PMC4406834 25909041

[aps370024-bib-0018] Chau, J. H. , W. A. Rahfeldt , and R. G. Olmstead . 2018. Comparison of taxon‐specific versus general locus sets for targeted sequence capture in plant phylogenomics. Applications in Plant Sciences 6(3): e1032. 29732262 10.1002/aps3.1032 PMC5895190

[aps370024-bib-0019] Cronn, R. , B. J. Knaus , A. Liston , P. J. Maughan , M. Parks , J. V. Syring , and J. Udall . 2012. Targeted enrichment strategies for next‐generation plant biology. American Journal of Botany 99(2): 291–311. 22312117 10.3732/ajb.1100356

[aps370024-bib-0020] De Vega, J. J. , S. Ayling , M. Hegarty , D. Kudrna , J. L. Goicoechea , Å. Ergon , O. A. Rognli , et al. 2015. Red clover (*Trifolium pratense* L.) draft genome provides a platform for trait improvement. Scientific Reports 5(1): 17394. 26617401 10.1038/srep17394 PMC4663792

[aps370024-bib-0021] Dizkirici, A. , M. Ekici , and Z. Kaya . 2014. Comparative molecular phylogenetics of *Astragalus* L. sections from Turkey with New World *Astragalus* species using nrDNA ITS sequences. Plant Systematics and Evolution 300: 163–175.

[aps370024-bib-0022] Doyle, J. J. 2022. Defining coalescent genes: Theory meets practice in organelle phylogenomics. Systematic Biology 71(2): 476–489. 34191012 10.1093/sysbio/syab053

[aps370024-bib-0023] Duan, L. , C. Su , J. Wen , Y. W. Ji , Y. Jiang , T. Zhang , J. L. Charboneau , et al. 2024. New insights into the phylogenetic relationships of tribe Astragaleae (Fabaceae subfamily Papilionoideae) and *Astragalus*—the largest genus of angiosperm. Biological Diversity 1(3–4): 136–146.

[aps370024-bib-0024] Eserman, L. A. , S. K. Thomas , E. E. Coffey , and J. H. Leebens‐Mack . 2021. Target sequence capture in orchids: Developing a kit to sequence hundreds of single‐copy loci. Applications in Plant Sciences 9(7): e11416. 34336404 10.1002/aps3.11416 PMC8312744

[aps370024-bib-0025] Ewels, P. , M. Magnusson , S. Lundin , and M. Käller . 2016. MultiQC: Summarize analysis results for multiple tools and samples in a single report. Bioinformatics 32(19): 3047–3048. 27312411 10.1093/bioinformatics/btw354 PMC5039924

[aps370024-bib-0026] Faircloth, B. C. , J. E. McCormack , N. G. Crawford , M. G. Harvey , R. T. Brumfield , and T. C. Glenn . 2012. Ultraconserved elements anchor thousands of genetic markers spanning multiple evolutionary timescales. Systematic Biology 61(5): 717–726. 22232343 10.1093/sysbio/sys004

[aps370024-bib-0027] Folk, R. A. , J. L. Charboneau , M. Belitz , T. Singh , H. R. Kates , D. E. Soltis , P. S. Soltis , et al. 2024. Anatomy of a mega‐radiation: Biogeography and niche evolution in *Astragalus* . American Journal of Botany 111(3): e16299. 38419145 10.1002/ajb2.16299

[aps370024-bib-0028] Forrest, L. L. , M. L. Hart , M. Hughes , H. P. Wilson , K. F. Chung , Y. H. Tseng , and C. A. Kidner . 2019. The limits of Hyb‐Seq for herbarium specimens: Impact of preservation techniques. Frontiers in Ecology and Evolution 7: e439.

[aps370024-bib-0029] González‐Domínguez, J. , and B. Schmidt . 2016. ParDRe: Faster parallel duplicated reads removal tool for sequencing studies. Bioinformatics 32(10): 1562–1564. 26803159 10.1093/bioinformatics/btw038

[aps370024-bib-0030] Granados Mendoza, C. , M. Jost , E. Hágsater , S. Magallón , C. van den Berg , E. M. Lemmon , A. R. Lemmon , et al. 2020. Target nuclear and off‐target plastid hybrid enrichment data inform a range of evolutionary depths in the orchid genus *Epidendrum* . Frontiers in Plant Science 10: e1761. 10.3389/fpls.2019.01761 PMC7000662 32063915

[aps370024-bib-0031] Johnson, M. G. , E. M. Gardner , Y. Liu , R. Medina , B. Goffinet , A. J. Shaw , N. J. C. Zerega , and N. J. Wickett . 2016. HybPiper: Extracting coding sequence and introns for phylogenetics from high‐throughput sequencing reads using target enrichment. Applications in Plant Sciences 4(7): e1600016. 10.3732/apps.1600016 PMC4948903 27437175

[aps370024-bib-0032] Johnson, M. G. , L. Pokorny , S. Dodsworth , L. R. Botigué , R. S. Cowan , A. Devault , W. L. Eiserhardt , et al. 2019. A universal probe set for targeted sequencing of 353 nuclear genes from any flowering plant designed using k‐medoids clustering. Systematic Biology 68(4): 594–606. 30535394 10.1093/sysbio/syy086 PMC6568016

[aps370024-bib-0033] Kalyaanamoorthy, S. , B. Q. Minh , T. K. Wong , A. Von Haeseler , and L. S. Jermiin . 2017. ModelFinder: Fast model selection for accurate phylogenetic estimates. Nature Methods 14(6): 587–589. 28481363 10.1038/nmeth.4285 PMC5453245

[aps370024-bib-0034] Kates, H. R. , B. C. O'Meara , R. LaFrance , G. W. Stull , E. K. James , S. Y. Liu , Q. Tian , et al. 2024. Shifts in evolutionary lability underlie independent gains and losses of root‐nodule symbiosis in a single clade of plants. Nature Communications 15(1): 4262. 10.1038/s41467-024-48036-3 PMC11130336 38802387

[aps370024-bib-0035] Kazempour Osaloo, S. H. , A. A. Maassoumi , and N. Murakami . 2005. Molecular systematics of the Old World *Astragalus* (Fabaceae) as inferred from nrDNA ITS sequence data. Brittonia 57(4): 367–381.

[aps370024-bib-0036] LPWG (The Legume Phylogeny Working Group) . 2017. A new subfamily classification of the Leguminosae based on a taxonomically comprehensive phylogeny: The Legume Phylogeny Working Group (LPWG). Taxon 66(1): 44–77.

[aps370024-bib-0037] Mai, U. , and S. Mirarab . 2018. TreeShrink: Fast and accurate detection of outlier long branches in collections of phylogenetic trees. BMC Genomics 19: 23–40. 29745847 10.1186/s12864-018-4620-2 PMC5998883

[aps370024-bib-0038] Mandel, J. R. , R. B. Dikow , V. A. Funk , R. R. Masalia , S. E. Staton , A. Kozik , R. W. Michelmore , et al. 2014. A target enrichment method for gathering phylogenetic information from hundreds of loci: An example from the Compositae. Applications in Plant Sciences 2(2): e1300085. 10.3732/apps.1300085 PMC4103609 25202605

[aps370024-bib-0039] Matsushima, R. , Y. Hu , K. Toyoda , Sodmergen , and W. Sakamoto . 2008. The model plant *Medicago truncatula* exhibits biparental plastid inheritance. Plant and Cell Physiology 49(1): 81–91. 18065422 10.1093/pcp/pcm170

[aps370024-bib-0040] Maylandt, C. , A. Seidl , P. Kirschner , S. Pfanzelt , G. Király , B. Neuffer , F. R. Blattner , et al. 2024. Phylogeography of the Euro‐Siberian steppe plant *Astragalus austriacus*: Late Pleistocene climate fluctuations fuelled formation and expansion of two main lineages from a Pontic‐Pannonian area of origin. Perspectives in Plant Ecology, Evolution and Systematics 64: e125800.

[aps370024-bib-0041] McKain, M. , and M. Wilson . 2017. mrmck ain/Fast‐Plast: Fast‐Plast v.1.2.6 (Version v.1.2.6). Available at Zenodo repository: 10.5281/zenodo.973887 [posted 23 September 2017; accessed 1 February 2024].

[aps370024-bib-0042] McKain, M. R. , M. G. Johnson , S. Uribe‐Convers , D. Eaton , and Y. Yang . 2018. Practical considerations for plant phylogenomics. Applications in Plant Sciences 6(3): e1038. 29732268 10.1002/aps3.1038 PMC5895195

[aps370024-bib-0043] Minh, B. Q. , H. A. Schmidt , O. Chernomor , D. Schrempf , M. D. Woodhams , A. Von Haeseler , and R. Lanfear . 2020. IQ‐TREE 2: New models and efficient methods for phylogenetic inference in the genomic era. Molecular Biology and Evolution 37(5): 1530–1534. 32011700 10.1093/molbev/msaa015 PMC7182206

[aps370024-bib-0044] Moonlight, P. W. , L. Baldaszti , D. Cardoso , A. Elliott , T. Särkinen , and S. Knapp . 2024. Twenty years of big plant genera. Proceedings of the Royal Society B, Biological Sciences 291(2023): e20240702. 10.1098/rspb.2024.0702 PMC11285793 38808446

[aps370024-bib-0045] Morales‐Briones, D. F. , A. Liston , and D. C. Tank . 2018. Phylogenomic analyses reveal a deep history of hybridization and polyploidy in the Neotropical genus *Lachemilla* (Rosaceae). New Phytologist 218(4): 1668–1684. 29604235 10.1111/nph.15099

[aps370024-bib-0046] Morales‐Briones, D. F. , B. Gehrke , C.‐H. Huang , A. Liston , M. Hong , H. E. Marx , D. C. Tank , and Y. Yang . 2022. Analysis of paralogs in target enrichment data pinpoints multiple ancient polyploidy events in *Alchemilla* s.l. (Rosaceae). Systematic Biology 71(1): 190–207. 10.1093/sysbio/syab032 PMC8677558 33978764

[aps370024-bib-0047] Ning, W. , H. M. Meudt , and J. A. Tate . 2024. A roadmap of phylogenomic methods for studying polyploid plant genera. Applications in Plant Sciences 12(4): e11580. 39184196 10.1002/aps3.11580 PMC11342234

[aps370024-bib-0048] Pezzini, F. F. , G. Ferrari , L. L. Forrest , M. L. Hart , K. Nishii , and C. A. Kidner . 2023. Target capture and genome skimming for plant diversity studies. Applications in Plant Sciences 11(4): e11537. 37601316 10.1002/aps3.11537 PMC10439825

[aps370024-bib-0049] Podlech, D. , and S. Zarre . 2013. A taxonomic revision of the genus Astragalus L. (Leguminosae) in the Old World. Naturhistorisches Museum Wien, Vienna, Austria.

[aps370024-bib-0050] POWO . 2024. Plants of the World Online. Facilitated by the Royal Botanic Gardens, Kew. Website https://powo.science.kew.org/ [accessed 4 September 2024].

[aps370024-bib-0051] Rajora, O. P. , and J. D. Mahon . 1995. Paternal plastid DNA can be inherited in lentil. Theoretical and Applied Genetics 90: 607–610. 24174016 10.1007/BF00222122

[aps370024-bib-0052] Ranwez, V. , E. J. Douzery , C. Cambon , N. Chantret , and F. Delsuc . 2018. MACSE v2: Toolkit for the alignment of coding sequences accounting for frameshifts and stop codons. Molecular Biology and Evolution 35(10): 2582–2584. 30165589 10.1093/molbev/msy159 PMC6188553

[aps370024-bib-0053] Sanderson, M. J. , and M. F. Wojciechowski . 1996. Diversification rates in a temperate legume clade: Are there “so many species” of *Astragalus* (Fabaceae)? American Journal of Botany 83(11): 1488–1502.

[aps370024-bib-0054] Shaw, J. , H. L. Shafer , O. R. Leonard , M. J. Kovach , M. Schorr , and A. B. Morris . 2014. Chloroplast DNA sequence utility for the lowest phylogenetic and phylogeographic inferences in angiosperms: The tortoise and the hare IV. American Journal of Botany 101(11): 1987–2004. 25366863 10.3732/ajb.1400398

[aps370024-bib-0055] Siniscalchi, C. M. , O. Hidalgo , L. Palazzesi , J. Pellicer , L. Pokorny , O. Maurin , I. J. Leitch , et al. 2021. Lineage‐specific vs. universal: A comparison of the Compositae1061 and Angiosperms353 enrichment panels in the sunflower family. Applications in Plant Sciences 9(7): e11422. 10.1002/aps3.11422 PMC8312747 34336403

[aps370024-bib-0056] Small, R. L. , R. C. Cronn , and J. F. Wendel . 2004. Use of nuclear genes for phylogeny reconstruction in plants. Australian Systematic Botany 17(2): 145–170.

[aps370024-bib-0057] Soltis, D. E. , and R. K. Kuzoff . 1995. Discordance between nuclear and chloroplast phylogenies in the *Heuchera* group (Saxifragaceae). Evolution 49(4): 727–742. 28565145 10.1111/j.1558-5646.1995.tb02309.x

[aps370024-bib-0058] Soltis, D. E. , P. S. Soltis , D. R. Morgan , S. M. Swensen , B. C. Mullin , J. M. Dowd , and P. G. Martin . 1995. Chloroplast gene sequence data suggest a single origin of the predisposition for symbiotic nitrogen fixation in angiosperms. Proceedings of the National Academy of Sciences, USA 92(7): 2647–2651. 10.1073/pnas.92.7.2647 PMC42275 7708699

[aps370024-bib-0059] Su, C. , L. Duan , P. Liu , J. Liu , Z. Chang , and J. Wen . 2021. Chloroplast phylogenomics and character evolution of eastern Asian *Astragalus* (Leguminosae): Tackling the phylogenetic structure of the largest genus of flowering plants in Asia. Molecular Phylogenetics and Evolution 156: e107025. 10.1016/j.ympev.2020.107025 33271371

[aps370024-bib-0060] Tang, H. , V. Krishnakumar , S. Bidwell , B. Rosen , A. Chan , S. Zhou , L. Gentzbittel , et al. 2014. An improved genome release (version Mt4. 0) for the model legume *Medicago truncatula* . BMC Genomics 15: e312. 10.1186/1471-2164-15-312 PMC4234490 24767513

[aps370024-bib-0061] Vargas, O. M. , M. Heuertz , S. A. Smith , and C. W. Dick . 2019. Target sequence capture in the Brazil nut family (Lecythidaceae): Marker selection and in silico capture from genome skimming data. Molecular Phylogenetics and Evolution 135: 98–104. 30818022 10.1016/j.ympev.2019.02.020

[aps370024-bib-0062] Varshney, R. K. , C. Song , R. K. Saxena , S. Azam , S. Yu , A. G. Sharpe , S. Cannon , et al. 2013. Draft genome sequence of chickpea (*Cicer arietinum*) provides a resource for trait improvement. Nature Biotechnology 31(3): 240–246. 10.1038/nbt.2491 23354103

[aps370024-bib-0063] Vatanparast, M. , A. Powell , J. J. Doyle , and A. N. Egan . 2018. Targeting legume loci: A comparison of three methods for target enrichment bait design in Leguminosae phylogenomics. Applications in Plant Sciences 6(3): e1036. 29732266 10.1002/aps3.1036 PMC5895186

[aps370024-bib-0064] Welsh, S. L. 2007. North American species of *Astragalus* Linnaeus (Leguminosae): A taxonomic revision. Monte L. Bean Life Science Museum, Brigham Young University, Provo, Utah, USA.

[aps370024-bib-0065] Wojciechowski, M. F. , M. Lavin , and M. J. Sanderson . 2004. A phylogeny of legumes (Leguminosae) based on analysis of the plastid *matK* gene resolves many well‐supported subclades within the family. American Journal of Botany 91(11): 1846–1862. 21652332 10.3732/ajb.91.11.1846

[aps370024-bib-0066] Yang, Y. , M. J. Moore , S. F. Brockington , D. E. Soltis , G. K. S. Wong , E. J. Carpenter , and Y. Zhang . 2015. Dissecting molecular evolution in the highly diverse plant clade Caryophyllales using transcriptome sequencing. Molecular Biology and Evolution 32(8): 2001–2014. 25837578 10.1093/molbev/msv081 PMC4833068

[aps370024-bib-0067] Yardeni, G. , J. Viruel , M. Paris , J. Hess , C. Groot Crego , M. de La Harpe , N. Rivera , et al. 2022. Taxon‐specific or universal? Using target capture to study the evolutionary history of rapid radiations. Molecular Ecology Resources 22(3): 927–945. 34606683 10.1111/1755-0998.13523 PMC9292372

[aps370024-bib-0068] Záveská, E. , C. Maylandt , O. Paun , C. Bertel , B. Frajman , P. Schönswetter , and Steppe Consortium . 2019. Multiple auto‐and allopolyploidisations marked the Pleistocene history of the widespread Eurasian steppe plant *Astragalus onobrychis* (Fabaceae). Molecular Phylogenetics and Evolution 139: e106572. 10.1016/j.ympev.2019.106572 31351183

[aps370024-bib-0069] Zhang, C. , and S. Mirarab . 2022. ASTRAL‐Pro 2: Ultrafast species tree reconstruction from multi‐copy gene family trees. Bioinformatics 38: 4949–4950. 36094339 10.1093/bioinformatics/btac620

[aps370024-bib-0070] Zhang, C. , R. Nielsen , and S. Mirarab . 2025. CASTER: Direct species tree inference from whole‐genome alignments. Science 387: eadk9688. 39847611 10.1126/science.adk9688 PMC12038793

[aps370024-bib-0071] Zhang, Q. , Y. Liu , and Sodmergen . 2003. Examination of the cytoplasmic DNA in male reproductive cells to determine the potential for cytoplasmic inheritance in 295 angiosperm species. Plant and Cell Physiology 44(9): 941–951. 14519776 10.1093/pcp/pcg121

[aps370024-bib-0072] Zhao, Y. , R. Zhang , K. W. Jiang , J. Qi , Y. Hu , J. Guo , R. Zhu , et al. 2021. Nuclear phylotranscriptomics and phylogenomics support numerous polyploidization events and hypotheses for the evolution of rhizobial nitrogen‐fixing symbiosis in Fabaceae. Molecular Plant 14(5): 748–773. 33631421 10.1016/j.molp.2021.02.006

[aps370024-bib-0073] Zimmer, E. A. , and J. Wen . 2015. Using nuclear gene data for plant phylogenetics: Progress and prospects II. Next‐gen approaches. Journal of Systematics and Evolution 53(5): 371–379.

