# Supplementary material for: Astragalean819: An Astragalean clade‐specific bait set to resolve phylogenetic relationships in Astragalus
Source: Appl Plant Sci. 2025 Oct 3;13(5):e70024. doi: 10.1002/aps3.70024 (PMC12542808; doi:10.1002/aps3.70024)
Supplement: Supplementary file 2 — Appendix S4: Variation in the number of raw reads sequenced from each specimen according to specimen age. The dashed line represents the trend line (R² = 0.078). Appendix S5: Summary of plastome assembly statistics. Appendix S6: AMAS statistics produced from raw homologous sequences. [file APS3-13-e70024-s002.pptx]

## Slide 1
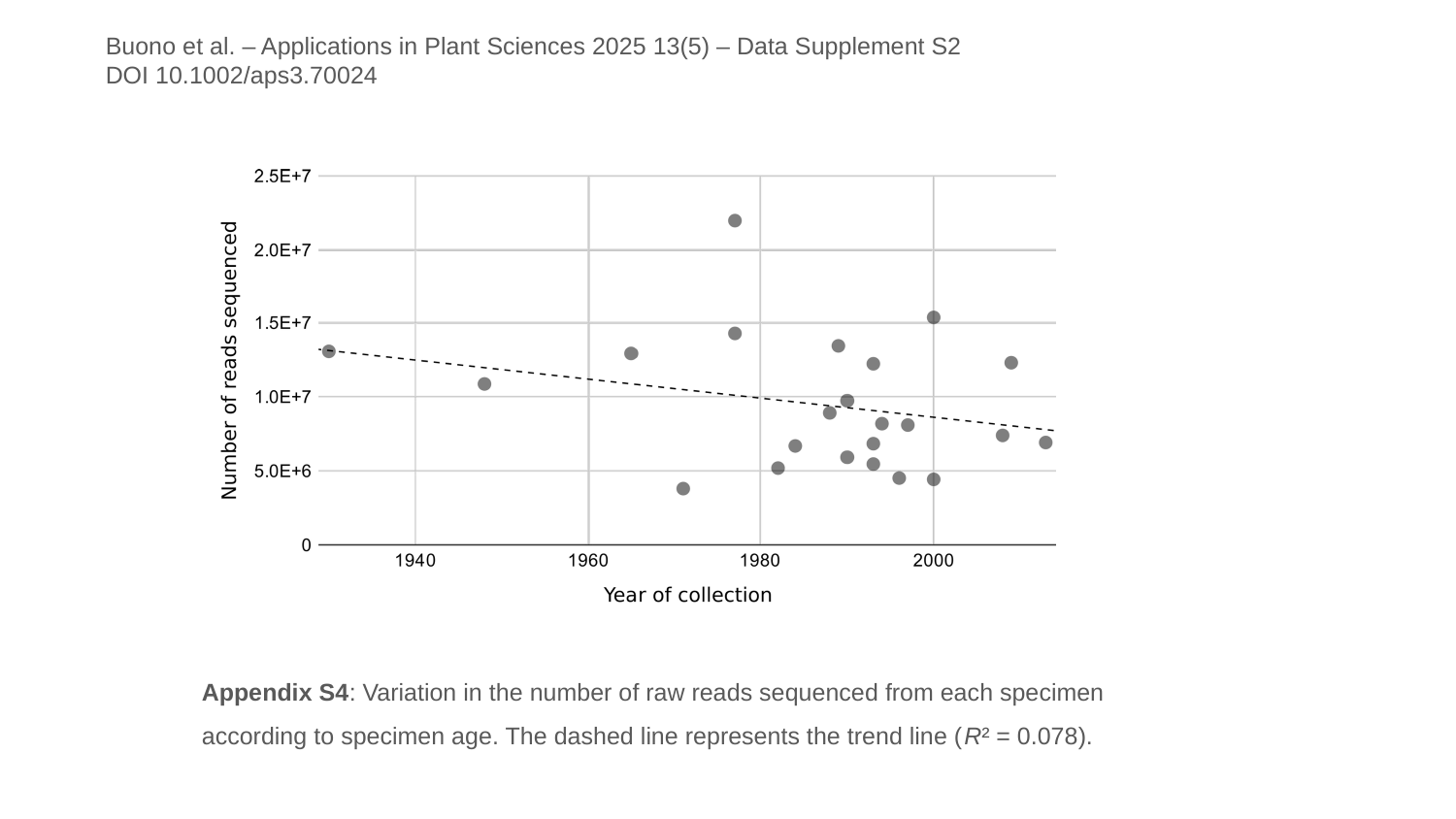

Buono et al. – Applications in Plant Sciences 2025 13(5) – Data Supplement S2
DOI 10.1002/aps3.70024
Appendix S4: Variation in the number of raw reads sequenced from each specimen according to specimen age. The dashed line represents the trend line (R² = 0.078).
